# Linking gender, climate change and security in the Pacific Islands Region: A systematic review

**DOI:** 10.1007/s13280-022-01813-0

**Published:** 2022-12-12

**Authors:** Elise Howard

**Affiliations:** Australian National University, Coombs Building, 9 Fellows Road, Acton, Canberra, ACT 2601 Australia

**Keywords:** Climate change, Gender, Inequality, Pacific, Security, Systematic review

## Abstract

This systematic review aims to address gaps in understanding how concepts of gender, climate change and security are given meaning and linked in empirical scholarship within the Pacific Islands Region. The review assesses the 53 articles returned through Web of Science, SCOPUS and ProQuest databases that are derived from empirical research and refer to gender, climate change and security. The findings indicate that this is an emerging topic in a region that is one of the most vulnerable to climate change across the globe. Most frequently gender analysis is given superficial treatment; there is limited literature that connects gendered vulnerabilities to historical legacies and structural inequalities; and women’s critical roles that create security are often overlooked and devalued. The review indicates that greater work is needed to question perceived threats to security and to reveal how climate change, gendered institutions, systems and spaces, historical legacies and politics interact to construct security in the Pacific Islands Region.

## Introduction

This review aims to address the gaps in understanding how concepts of gender, climate change and security are given meaning and linked in empirical scholarship in the Pacific Islands Region. How security and gender are defined and interpreted—and how these concepts are linked to climate change—matters as this shapes policy responses, the issues that are considered or overlooked within those responses, and who will most benefit. Climate security is a contested concept, both in the sense of the security issues or threats that may be ‘multiplied’ due to climate change, and the measures that need to be put in place to ensure security in the face of climate change impacts (Rasheed 2022). Taking a gendered lens to the links between climate change and security can enable greater understanding of how multiple crises and points of inequality are interconnected (Tanyag and True 2019; de Jonge Oudraat and Brown 2022), what is needed to address gender inequality concurrently with climate risks (UNEP et al. 2020) and to improve the effectiveness of climate adaptation actions designed to promote peace and stability (IPCC 2022).

Male and western voices dominate within the institutions that have the most agency in constructing both the problem and the proposed solutions to climate security (Oswald Spring 2009; Detraz 2015; de Jonge Oudraat and Brown 2022; Rasheed 2022). In this context, minimal attention has been paid to gender inequality as a key source of insecurity (Tanyag 2020; UNEP et al. 2020; de Jonge Oudraat and Brown 2022). While there is broad-scale recognition that climate change impacts will disproportionately affect the most vulnerable groups in society (Arora-Jonsson 2011; Kaijser and Kronsell 2013; Adger et al. 2014; Rydstrom and Kinnvall 2019; IPCC 2022), women’s vulnerability has been overemphasised in relation to their agency (Arora-Jonsson 2011), and there is a lack of context-specific understandings on how gender inequalities, climate change and security are linked (UNEP et al. 2020).

The geographically and culturally diverse Pacific Islands Region is a site where climate change impacts are being experienced frequently and intensely. Low-lying atolls are particularly at risk of storm surge and sea level rise, and all island nations are prone to extended droughts, warming sea temperatures, changing rainfall patterns and increased frequency and intensity of cyclones (Mycoo et al. 2022). In this region, climate change and security have been linked due concerns about projected resource shortages, increasing mobility, and population movement within nations and across the region (Barnett 2009; Chand and Taupo 2020). Women are often depicted as the most vulnerable group within the region without attention to diversity in women’s experiences or intersectional forms of status (Jolly 2020). Yet on the whole, women are under-represented in leadership and decision-making in political and policy spaces (Underhill-Sem et al. 2016; Baker 2019), and rates of gender-based violence are higher than the global average (Pacific Women 2020). In this context, certain perspectives are likely to be privileged over others due to gendered hierarchies of knowledge, legitimacy and voice, meaning responses have the potential to worsen or reinforce inequalities (Eriksen et al. 2015; de Jonge Oudraat and Brown 2022).

Approaches to researching climate change in the Pacific Islands Region are varied, and so analysis to determine how concepts of gender, climate change and security are being linked is complex. There are some systematic reviews in relation to climate change adaptation in the Pacific (McNamara and Buggy 2017; Klöck and Nunn 2019; Weir 2020), and in relation to climate security (Tangney et al. 2021) yet these reviews did not consider how gender is articulated and applied within climate change research. There are reviews of how women are positioned within policies dealing with disaster and climate change adaptation (Aipira et al. 2017), and complementary scholarship, particularly in the fisheries sector which demonstrates that policy and practice tends to ‘tinker’ with rather than transform gender relations (Lawless et al. 2021, 2022; Mangubhai et al. 2022), yet gaps remain in understanding how climate change, security and gender are connected.

This systematic review aims to address this gap by mapping the current literature landscape to understand the interaction across gender, climate change and security in empirical research, how these concepts have been interpreted and applied, thematic areas, and areas for future research. It commences by sharing conceptual frameworks to illustrate the varied meanings and approaches to climate security and gender. Secondly, the article shares the systematic review methodology. Thirdly, the article provides a descriptive overview of how security and gender are given meaning and linked to climate change in empirical scholarship in the Pacific Islands Region.

The review finds that, most frequently, gender analysis within the context of climate change and security is given superficial treatment, where gender is equated with women and women are included as research participants, or a gender division of labour within a community is undertaken. While women are frequently represented as most vulnerable to climate change, there is limited literature that grounds these vulnerabilities within historical legacies that have naturalised divisions between public and private issues, domestic and ‘productive’ work or formal and ‘informal’ economies; constructions of masculinity and femininity; or the political contestations that lead to structural inequalities and insecurities. At the same time, women’s critical roles that create security are often overlooked and devalued due to the gendered nature of institutions. Greater attention is needed to context-specific understandings of how gendered institutions, systems and spaces, politics, historical legacies, climate change impacts and differential experiences of security are connected.

### Security, climate change and gender

The concept of climate security is gaining traction in policy making in the Pacific Islands Region. In 2018 and again in 2022, the Pacific Islands Forum, the pre-eminent regional policy organisation, directly linked concerns between climate change and security (PIFS 2018, 2022). In 2020, the United Nations announced a climate security project for Kiribati, Tuvalu and the Marshall Islands, that aims to respond to the ‘cascading fragility and instability risks’ (UNDP 2020, para 3) posed by climate change. Yet continued debates on the links between climate change and security (Barnett 2009; Adger 2011; Gemenne et al. 2014; Rasheed 2022) illustrate that it is important to interrogate the ways in which the meaning of security is constructed and linked to climate change (McDonald 2021).

Security is a contested and temporal concept, influenced by the values and preoccupations of different political communities and geo-political tensions at particular points in time (McDonald 2021). The question of whose security is under consideration (or the referent object) will influence the way that the relationship between climate change and security is conceived and shape perceptions of appropriate responses (Oswald Spring 2009; Detraz 2015; McDonald 2021). Initial links between climate change and security prioritised international or national security as the referent object, however, contemporary approaches centre human security as the referent object, that is environmental, ecological, social, cultural and human rights, freedoms, and agency to act at the individual and community level (Adger et al. 2014). Through a human security lens, climate security can be defined as the condition where people, communities, states and international organisations have the capacity to manage the stresses driven by climate change, the necessary options to respond to the threats to their human, environmental and social well-being imposed by climate change, and have the freedom and agency to exercise these options (Adger 2011; Dellmuth 2018).

The links between climate change and security are understood through frameworks that link threats, vulnerabilities and means as illustrated in Table 1. Threats are clearly identifiable, immediate concerns which require decisive and quick responses. Means are the manner in which security is to be preserved in response to the threat, largely achieved through climate change mitigation by reducing emissions or adaptation to reduce exposure to climate change (Oswald Spring 2009; Detraz 2015; Eriksen et al. 2015; McDonald 2021). Vulnerability comprises three elements: risks that create exposure to harm, the sensitivity or susceptibility to harm, and the agency, capacity or assets to cope or reduce the level of risks (Adger 2006; McLaughlin and Dietz 2008).

**Table 1:** Climate security frameworks link threats, vulnerabilities and means that vary according to the referent object, developed with reference to Oswald Spring (2009), Adger (2011), Detraz (2015) and McDonald (2021)

Referent object	International security	National scurity	Human security
Threats	Unmanaged international migration as a threat to national borders or international resource-driven violent conflicts due to sea level rise, natural disasters, or resource scarcity	Localised violent conflicts and internal population movements, resource ‘wars’ or territorial boundaries, and risks to critical infrastructures and energy security due to sea level rise, natural disasters, or resource scarcity	Compromised well-being due to shortages in basic material needs (food, water, health and education), and non-material needs (liberties, dignities, freedom from fear, culture, identity and a sense of place)
Vulnerability	Capacity to reduce global climate impacts, manage population movements, and ‘fragile state’ breakdown	Capacity to reduce risk exposure, stimulate adaptation, protect public goods and infrastructures, and regulate land use and population movements	Position within unequal political, economic and social structures
Means	International negotiation and cooperation, climate change mitigation and adaptation	Climate change adaptation and sustainable development, migration laws, early warning systems, planning and responses in times of disaster	Climate change adaptation and sustainable development. Inclusive and transparent governance, education, health, sanitation. Address inequality and marginalisation

These concepts are neither politically nor gender neutral (Oswald Spring 2009; Detraz 2015). For example, vulnerability is influenced by unequal structures, such as a person’s social, cultural, economic status, physical location and degree of political marginalisation (Eriksen et al. 2015), with varying possibilities for individual or collective agency to act within these structures (McLaughlin and Dietz 2008). Moreover, threats tend to focus on environmental change rather than the root causes of environmental degradation, political economies or power hierarchies (Robbins 2012). Finally, adaptation efforts are influenced by power imbalances, which can mean that actions taken purportedly to reduce vulnerabilities in fact increase inequalities in experiences of climate change, or maladaptation (Barnett and O’Neill 2010).

More critical approaches to analysing security highlight the male domination in the institutions that have had the most agency in constructing the problem of climate security, that is, United Nations forums, science academies and militaries (Oswald Spring 2009; Detraz 2015); the legacies of colonisation for maintaining power hierarchies in global forums and shaping security circumstances in different locations (Ratuva 2019); and the dismissal of family violence, rape during crisis, or inequality and poverty as key security concerns (Tickner 2006; Wonders 2018). Feminist approaches broaden understandings of security and reframe ‘in-kind’, ‘private’ or ‘informal’ work such as caring and household food provision, as a key source of social stability and security (Robinson 2011; Teaiwa 2021). Critical approaches also highlight non-dominant worldviews and place religion, cosmology or social networks as a key source of ontological security (security of ‘being’) during times of existential crisis (Shani 2017; Farbotko 2019).

Given the diverse understandings of these concepts and the links between climate change and security, it is important to recognise that security is a social and political construction which will be framed differently by different actors at different points in time (Detraz 2015; McDonald 2021). This review therefore sought to understand how the meaning of security in the context of climate change is constructed in empirical scholarship across the Pacific Islands Region whether security was merely mentioned, inferred through threats and vulnerabilities, defined, described or critically analysed. Secondly, the review sought to understand what types of approaches are taken to analyse gender within this scholarship.

**Fig. 1:**
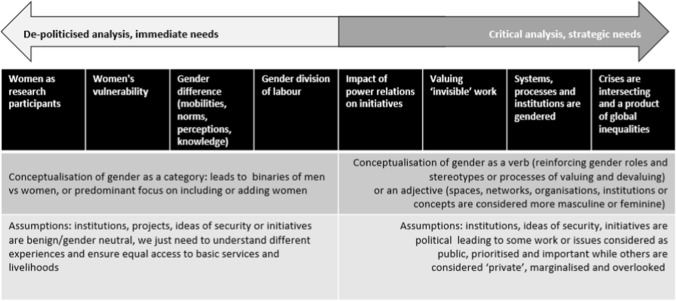
Addressing gender in climate change research occurs along a spectrum ranging from including women as research participants through to considering the root causes that interact to create multiple crises. Developed with reference to Rocheleau et al. (1996), Peterson (2005), Tickner (2006), Kronsell (2010), Robinson (2011), Hansen (2014), Enloe (2014), Detraz (2015), Elmhirst (2015), Runyan and Peterson (2015), Tanyag (2018) and Sundberg (2017)

Gender is a widely used and diversely applied concept. For this review, literature was categorised on a spectrum that ranged from an ‘adding women’ approach through to research that engages critically with the gendered inequalities that are created and sustained through unequal global arrangements (see Fig. 1).

At one end of the spectrum, gender is conceptualised as a category, and is considered to be ‘done’ when women are included in meetings, projects, consultations or data collection, some form of gender disaggregated analysis occurs, or research engages with differences between men and women and their differential capacities to cope with climate change. These approaches are often called ‘adding women’ to initiatives, organisations, policies or research and tend to be focussed on immediate and practical needs; informed by instrumental toolkits or checklists (Cornwall et al. 2007); and treat women as one category, who are disadvantaged as a whole relative to men, rather than the intersectional factors that produce inequality (Arora-Jonsson 2011; Kaijser and Kronsell 2013; Djoudi et al. 2016).

At the more critical end of the spectrum, gender is conceptualised as a process of ‘gendering’ or as an adjective such as ‘gendered organisations’. This scholarship recognises that processes of valuing masculine roles and spaces and devaluing feminine roles and spaces lead to hierarchies of legitimacy that create substantial inequalities. Some issues will be considered a priority and public, while others may be overlooked or dismissed as domestic or private.

For example, research that considers gender as a power relation highlights that differentials in power occur in across all contexts at household, community, national or global levels. Power is not something that is given or taken, but rather maintained and exercised through biased structures which lead to inequalities in perceived options for responding to and adapting to climate change (Kronsell 2019). Engaging with power is political and can be disruptive to the status quo, resulting in backlash, resistance or dismissal tactics. It is so often why gender is relabelled in friendlier terms such as ‘diversity’ and watered-down approaches that revert to ‘adding women’ (Ahmed 2017). Feminist political ecology approaches consider how spaces maintain and produce power in gender relations. As a simple example, separating women during menstruation to avoid pollution of men or surrounding environments is one means of using space to construct power in gender relations (Rocheleau et al. 1996; Sundberg 2017).

A further body of work aims to value women’s work and make women’s contributions visible. This scholarship aims to provide greater visibility to women’s experiences and contribution to society. While these approaches could be seen as ‘adding women’, they are differentiated by analysis of gender as a construct that shapes taken for granted notions of masculinity and femininity that are ascribed to social roles, social and cultural norms, social relations, organisations and institutions (Runyan and Peterson 2015). These approaches examine differential values placed on women and men’s work (Mohanty 2003; Peterson 2005; Runyan and Peterson 2015; Tanyag 2018) and expose how institutions and organisations are structured towards masculine bodies and values (Peterson 2005; Kronsell 2010; Enloe 2014).

Further along the spectrum, research explores how the gendered nature of institutions leads to women’s exclusion or subordination (see, for example, Carter and Howard 2020). Gendered institutional approaches are important in a field that is highly male dominated across all domains of research, negotiations, advocacy, policy development and implementation, due to the privileging of science and technological solutions to climate change (Djoudi et al. 2016). Scholars in this field reveal how climate policy and negotiations ‘reflect the gendered power, privilege and pre-occupation of most policy makers around the world’ (Nagel 2012, p. 470), are prone to elite capture (Eriksen et al. 2015, 2021) and constrain who can define what a security threat is, who is perceived as a victim and what counts as a security issue (Hoogensen and Stuvøy 2006; Oswald Spring 2009; Detraz 2017). Feminist political ecology approaches question the privileging of science knowledge and devaluing of custodian knowledges, as well as notions of ‘objectivity’ and ‘value free research’ (Rocheleau et al. 1996). These approaches consider how unequal processes, institutions, taken for granted social and cultural norms, relationships and hierarchies construct gendered access to decision making or funding or gendered differentials in experiences of crises such as climate change.

Finally, a body of work situates gendered experiences within intersecting crises, and the social, political and economic structures that exacerbate gender inequality. These approaches demonstrate that intersectional experiences of vulnerability are attributable to global political economy, inequalities in wealth accumulation and resource exploitation, capitalism and militarisation (Peterson 2005; Tickner 2006; True 2012; Enloe 2014; Runyan and Peterson 2015; Teaiwa 2021) and call on climate responses to address root causes rather than rely on technological solutions (Tanyag and True 2019). Feminist political ecology approaches question notions of ‘sustainable development’ particularly in capitalist contexts where ‘productive’ work is valued over ‘non-productive’ work such as health or care work, cultural or spiritual practices. In this field, analysis focuses on social–ecological relations to determine how social, political and economic arrangements *and* natural environments shape gender relations (Rocheleau et al. 1996; Sundberg 2017).

## Method

This systematic review followed the steps outlined in Pickering and Byrne (2014) with reference to the PRISMA checklist (PRISMA 2020). Initial literature searches returned few relevant results and were missing key authors who are well known in the field for their work to link gender, climate change and security (such as Bhagwan Rolls and Rolls 2019; George 2014, 2016). Search terms were tested and refined until a high level of confidence that the search had captured a large proportion of relevant literature was achieved. As highlighted earlier, security is a term that has typically excluded considerations of sexual violence or non-productive and caring work. Given the potential for this term to exclude studies that do not mention security but do consider links between sexual violence and droughts or disasters, the usage of climate change OR security rather than climate change AND security in the search string was important to capture these studies (see Table 2). The search strings differ slightly from each other due to syntax conventions on each database. Literature searches were undertaken on 14 January 2022 using Web of Science, SCOPUS and ProQuest databases.

**Table 2:** Search strings to capture a broad range of literature

Database	Search string	Results
Web of Science	(‘climate change’ OR adapt OR resili* OR security) AND (gender OR gender politics OR women OR inequal*) AND (‘Cook Islands’ OR Caledonia OR Fiji OR Guam OR Kiribati OR ‘Marshall Islands’ OR Nauru OR Niue OR Palau OR Papua OR Guinea OR Samoa OR Solomon OR Tonga OR Tuvalu OR Vanuatu OR Niue OR ‘Pacific Island*’ OR Oceania OR Melanesia OR Micronesia OR Polynesia)	466
SCOPUS:	ALL (({climate change} OR adapt OR resili* OR security) AND (gender OR gender AND politics OR women OR inequal*) AND ({Cook Islands} OR caledonia OR fiji OR guam OR kiribati OR ‘marshall AND islands’ OR nauru OR niue OR palau OR papua OR guinea OR samoa OR solomon OR tonga OR tuvalu OR vanuatu OR niue OR {Pacific Island*} OR oceania OR melanesia OR micronesia OR polynesia)) AND (LIMIT-TO (AFFILCOUNTRY, "Fiji") OR LIMIT-TO (AFFILCOUNTRY, "Federated States of Micronesia") OR LIMIT-TO (AFFILCOUNTRY, "Samoa") OR LIMIT-TO (AFFILCOUNTRY, "Papua New Guinea") OR LIMIT-TO (AFFILCOUNTRY, "Solomon Islands") OR LIMIT-TO (AFFILCOUNTRY, "Marshall Islands") OR LIMIT-TO (AFFILCOUNTRY, "Vanuatu") OR LIMIT-TO (AFFILCOUNTRY, "Guam") OR LIMIT-TO (AFFILCOUNTRY, "Kiribati") OR LIMIT-TO (AFFILCOUNTRY, "Tonga"))	136
ProQuest	((‘climate change’ OR adapt OR resili* OR security) AND (gender OR gender politics OR women OR inequal*) AND (loc.exact("Asia–Pacific region" OR "Oceania" OR "Papua New Guinea" OR "Vanuatu" OR "Pacific Islands" OR "Solomon Islands") AND at.exact("Article" OR "Dissertation/Thesis" OR "Book" OR "Country Report") AND subt.exact("political science" OR "sociology" OR "social sciences" OR "politics" OR "womens studies" OR "cultural anthropology" OR "covid-19" OR "studies" OR "international relations" OR "climate change" OR "minority & ethnic groups" OR "women" OR "families & family life") AND la.exact("ENG") AND pd (20170114–20220114))	538

Article titles were then reviewed manually to exclude irrelevant articles, such as political reviews or studies from other parts of the globe. All results were combined into an excel spreadsheet and duplicates removed, resulting in 195 articles. Then inclusion and exclusion criteria (see Table 3) were applied by reading abstracts and scanning articles to ensure the review was limited to empirical research yet broad enough to include diverse understandings of security. For example, studies that did not explicitly refer to security, but referred to gender-based violence, women’s mobility constraints, or considered how risk perceptions are gendered were included. This resulted in a final sample of 53 studies which were downloaded to a secure OneDrive folder.

**Table 3:** Inclusion and exclusion criteria

Inclusion criteria	Exclusion criteria
• Study considers gender or includes women • Article is based on historical or contemporary lived experiences or collected data with a community or multiple communities, policy makers or activists • Study located within the Pacific Islands Region as defined by the Pacific Islands Forum Secretariat (excluding Australian and NZ): Cook Islands, Federated States of Micronesia, Fiji, French Polynesia, Kiribati, Nauru, New Caledonia, New Zealand, Niue, Palau, Papua New Guinea, Republic of Marshall Islands, Samoa, Solomon Islands, Tonga, Tuvalu and Vanuatu • Refers to climate change risks, threats or security, conflicts, gender-based violence or constraints on women’s mobility; social or political effects; adaptation, resilience or vulnerability; or disaster assessment and response	• Theoretical articles, opinion pieces, systematic literature or desktop reviews • Countries outside of the Pacific Islands region • Studies with a primary focus on documenting climate science data • Duplicate articles based on the same empirical study

Article details were entered into a database including study location, rural or urban focus, publication year and format, methodology, climate risks, broad treatment of gender and mentions of security. In the next step, deductive coding in NVivo was undertaken using the framework provided at Table 1 to detail the treatment of security (named only, inferred or explicitly analysed), and types of security, threats, means and/or vulnerabilities in each article. Deductive coding was also applied for treatment of gender according to the spectrum in Fig. 1. This process was complemented by a memo to record observations about each article. Coding was then cross-checked with the initial database, in-text searches within NVivo and memos to develop themes and categories. Articles that engaged more substantially with gender as an analytical concept were then selected for in-depth review. An iterative process of memo writing on the key themes emerging through the literature enabled a final analysis.

Systematic reviews are a useful means of drawing together research from diverse disciplines to highlight the breadth and depth of research being undertaken, diversities and commonalities, the combinations of subjects studied and research gaps (Pickering and Byrne 2014). Systematic reviews, however, have several limitations, particularly the risk of missing literature. In the Pacific Islands Region, much knowledge and expertise is held outside of academic journals. This was addressed to some extent by using three databases for the searches, however, it is acknowledged that academic literature is exclusive of differing forms of communication and knowledge systems, moreover, the review is limited to English language articles only.

### Publication year and study locations

While no time limitations were placed on the searches, the earliest publication in this sample was 2010. More general climate change research, particularly scientific research, in the Pacific dates to the 1990s. The short time span covered in this sample may reflect that the links between climate change and security were discussed in significant forums from the early 2000s and there has been a relatively recent recognition of the social, political and gendered implications of climate change. The small number of articles in 2022 is due to the search being undertaken in January. While the end of the calendar year is a neater cut-off, articles published in January 2022 added more in-depth substance to this review and so were included (see Table 4).

**Table 4:** Publications per year and study locations

Study location	Publication year													
	2010	2011	2012	2013	2014	2015	2016	2017	2018	2019	2020	2021	2022	Country total
Tuvalu												1		1
French Polynesia										1				1
New Caledonia										1				1
Regional					1		1		1					3
Kiribati										1	1	2		4
Tonga										1	1	2		4
Papua New Guinea		1	1						1	1		2		6
Samoa	1				1						2	2		6
Solomon Islands		1			1				1	4	2	2		11
Fiji							1	1		3	1	6	2	14
Vanuatu							1	3	2	3	7	4		20
Total articles per year	1	2	1	0	3	0	3	4	5	9*	12^^^	11^#^	2	

*Total includes 1 article covering New Caledonia, Fiji, Vanuatu, Tonga, PNG, Solomon Islands and 1 article covering Solomon Islands and Kiribati

^^^Total includes 1 article covering Kiribati, Tonga and Samoa

^#^Total includes 1 article covering Fiji and Tonga, 1 article covering Solomon Islands, Vanuatu and Samoa and 1 article primarily focussed on PNG and including Fiji, Kiribati, Samoa, Solomons Islands, Tonga, Tuvalu and Vanuatu as secondary sites

Vanuatu was the most highly studied location (20 articles) in the sample due to several studies in response to the impacts of Cyclone Pam as well as an ARC project on Community-Based Adaptation (led by McNamara, Nunn and Watson) which had a number of study sites Vanuatu. Vanuatu is followed by Fiji (14 articles) which is highly represented due to the number of studies that considered the gendered impacts of cyclones Winston and Harold as well as work within the fisheries sector to improve visibility of women’s roles. Fiji is followed by Solomon Islands (11), Papua New Guinea (6), Samoa (6), Kiribati (4), Tonga (4), Regional studies (3), and French Polynesia (1), New Caledonia (1) and Tuvalu (1). Note some double counting has occurred because of multi-site studies (see Table 4).

### What type of security was named or how was security inferred?

Articles were reviewed to capture how security is given meaning and linked to climate change. Deductive coding based on the framework in Table 1 was used to identify whether security was given explicit treatment in the context of climate change, or could be inferred through means, threats and vulnerabilities. Coding was then grouped into overarching themes, and literatures sorted into common and more critical understandings of the links between climate change and security (see Table 5). While close to a third of the sample did explicitly consider types of security, the remaining articles highlight some important elements that could be considered within climate security frameworks.

**Table 5:** Summary themes on the links between climate change and security in existing empirical research

	Common understandings	Critical understandings
Security types	Food and nutritional security Energy security and poverty Water security	Women Peace and Security agenda (1325) Land tenure and forced evictions A 'sense' of security: affected by ontological security, identity, stress, grief, emotions, connection to place, spirituality, traditional beliefs or culture Sexual and reproductive health as a form of security
Threats	Cyclones, flood, sea level rise, displacement, drought	Discourses that lead to maladaptation Elite capture Poorly designed aid, policies or management of resource extraction Perceptions of risk and threats are gendered and affected by level of education, urban and rural locations. Reports on community risk perceptions may be affected by power differentials
Vulnerability	Geographic conditions, high rainfall, coastal areas, lack of infrastructure and government services	Poor governance, high levels of gender-based violence, inequalities, social norms that constrain women's mobility, lack of workload burden sharing with flow on effects for livelihoods, health, and food supply, lack of women’s representation in leadership and decision-making Legacies of colonisation
Means	Adaptation, livelihoods, perceptions and understanding as a pre-cursor to adaptation, traditional ecological knowledge or social networks as a means of resilience and adaptation	Caring work, food production and marketing underpin social capital and are a means of creating ontological security Experiences depend on intersectional forms of status, for example, migrant and displaced communities lack TEK and networks for adaptation Local risk perceptions may be shaped by spiritual knowledge

Within the sample, 17 articles had a significant focus on types of security in the context of climate change. This included seven articles that had a primary focus on food and nutritional security (Cleasby et al. 2014; Eriksson et al. 2017; Rabbitt et al. 2019; Savage et al. 2020; Wentworth et al. 2020; Cauchi et al. 2021; Mangubhai et al. 2021); three articles that sought to expand the Women, Peace and Security Agenda in the region to include climate change impacts as a human security issue (George 2014, 2016; Bhagwan Rolls and Rolls 2019); two articles that considered women’s roles in community resilience, with a particular emphasis on food security (Davila et al. 2021) or ontological security (Singh et al. 2022); one article on energy security (Sovacool et al. 2012) or energy poverty (Teariki et al. 2020); one article considering land tenure security and forced evictions (Day et al. 2021); one article on sexual and reproductive health responses in post-disaster periods which included the challenges of being considered as a ‘security’ issue (Beek et al. 2021); and one article on the gendered nature of village water committees and water security (Nelson et al. 2021). While this illustrates that the material aspects of human security, particularly food security, are being used to frame explicit treatments of security, this tends to be siloed. Literature that connects the non-material aspects of human security, such as dignity, freedom from fear or to exercise choice, with material needs such as education, livelihoods and food security was limited. This is not surprising as the connection between security and climate change is recent in policy and scholarship in the Pacific, while concepts such as food and water security have been engaged with explicitly through specialist studies for quite some time.

More often security was named but not defined or analysed (27 articles). This occurred where security was generally named as part of setting out the problem and the cross-cutting issues that would be exacerbated by climate change. In this sample, once again food security is the mostly commonly named type of security (18/27). In addition, a significant proportion of the sample was framed in terms of disaster response, including 16 articles that were written to review post-cyclone recovery.

A broad approach was important to capture the considerations that underpin understandings of the links between climate change and security. The literatures raised issues around perceptions of risk and threat as political and reflective of power dynamics. For example, the influence of power differentials on reporting of risk perceptions, and the ‘subtle processes through which are risks are normalised or prioritised according to social, cultural or political influences’ (Ensor et al. 2018, p. 1139) has a significant influence on ‘which risks are perceived as significant and how they are acted on’ (Ensor et al. 2018, p. 1139). These power differentials may occur within communities, or be apparent in the mismatch between community perceptions and top-down approaches (Lipset 2011; Pascoe et al. 2021). In some articles, this mismatch and dismissal of local worldviews or loss of culture was framed as one of the legacies of colonisation (Grant 2019; Wentworth 2020; Pascoe et al. 2021).

A number of studies highlighted the role of social capital as a security network, and traditional ecological knowledge (TEK) and care work as a means of creating social security (Rey et al. 2017; Balaei et al. 2019; Crichton et al. 2020; Clissold et al. 2020; Malherbe 2020; Senimoli et al. 2020; Chambers et al. 2021; Sahai et al. 2021; Singh et al. 2022). However, this should not be romanticised, nor the experience of living in the Pacific homogenised: while social capital and traditional ecological knowledge are important strengths for indigenous communities living on customary land, not all citizens or communities live in this situation (Chandra and Gaganis 2016; Nakamura and Kanemasu 2022).

The review also highlighted important threats to consider beyond climate change, such as the potential for discourse to cause greater problems than the actual climatic threats themselves (van der Ploeg 2020; Nef 2021), or the possibility that elite capture, poor regulation or poorly designed responses can lead to greater inequalities and divisions within communities (Buggy and McNamara 2016; Roberts 2019; Day et al. 2021; van der Ploeg et al. 2020; Westoby et al. 2020). Externally driven projects, whether for resource development or adaptation, can drive significant local fractions, and either reinforce existing power differentials within households and between families, or create new power structures (Buggy and McNamara 2016; Roberts 2019). Power differentials can lead to a lack of inclusive decision making as well as differences in access to material benefits such as housing, water tanks, employment or access to training opportunities that are intended to reduce vulnerabilities (Clissold et al. 2020; Westoby et al. 2020). Moreover, the review highlights the importance of mobility to livelihood and health security (Asugeni et al. 2019) which can be constrained by social norms that restrict women’s mobilities to local villages (Rabbit et al. 2019; Singh et al. 2022).

The links between gender-based violence and climate change were referred to in a number of articles, such as increases in violence during drought or post-disaster periods (George 2014, 2016; Chandra and Gaganis 2016; Mcleod 2018; Bhagwan Rolls and Rolls 2019; Clissold et al. 2020; van der Ploeg 2020; Webb 2020; Beek et al. 2021; Davila et al. 2021; Sahai 2021) yet the documentation of this is limited due to the sensitive nature and difficulties in researching this topic. Studies tended to note this was an issue, and shared women’s concerns about this (see in particular Mcleod et al. 2018) but did not seek to collect data specifically on incidences of violence.

### How are concepts of gender, climate change and security linked in empirical scholarship?

Articles were then reviewed to consider how gender was predominantly analysed (according to the spectrum at Fig. 1) and linked to climate change and security. The majority of articles (35) were classified at the uncritical end of the spectrum, with a brief mention of women only (3), including women as participants (13), predominantly reporting on or framing women in terms of vulnerabilities (6), or predominantly reporting on gender differences or the gender division of labour (13). While some articles noted the importance of recognising gender as power relations in conceptual frameworks, this was often not backed up in data analysis which instead reported on gender difference. There may be a number of reasons for this, such as the simplification of gender analysis into toolkits that are designed for use by practitioners with expertise in other fields, or the influence of donor agencies who require women to be included or a gender disaggregation of data. Alternatively, this may be due to the relatively recent focus on gendered experiences of climate change and the lack of attention to gender within the field of security studies. The remaining articles (18) were selected for more in-depth review to determine how gender, climate change and security are linked in empirical scholarship.

**Fig. 2:**
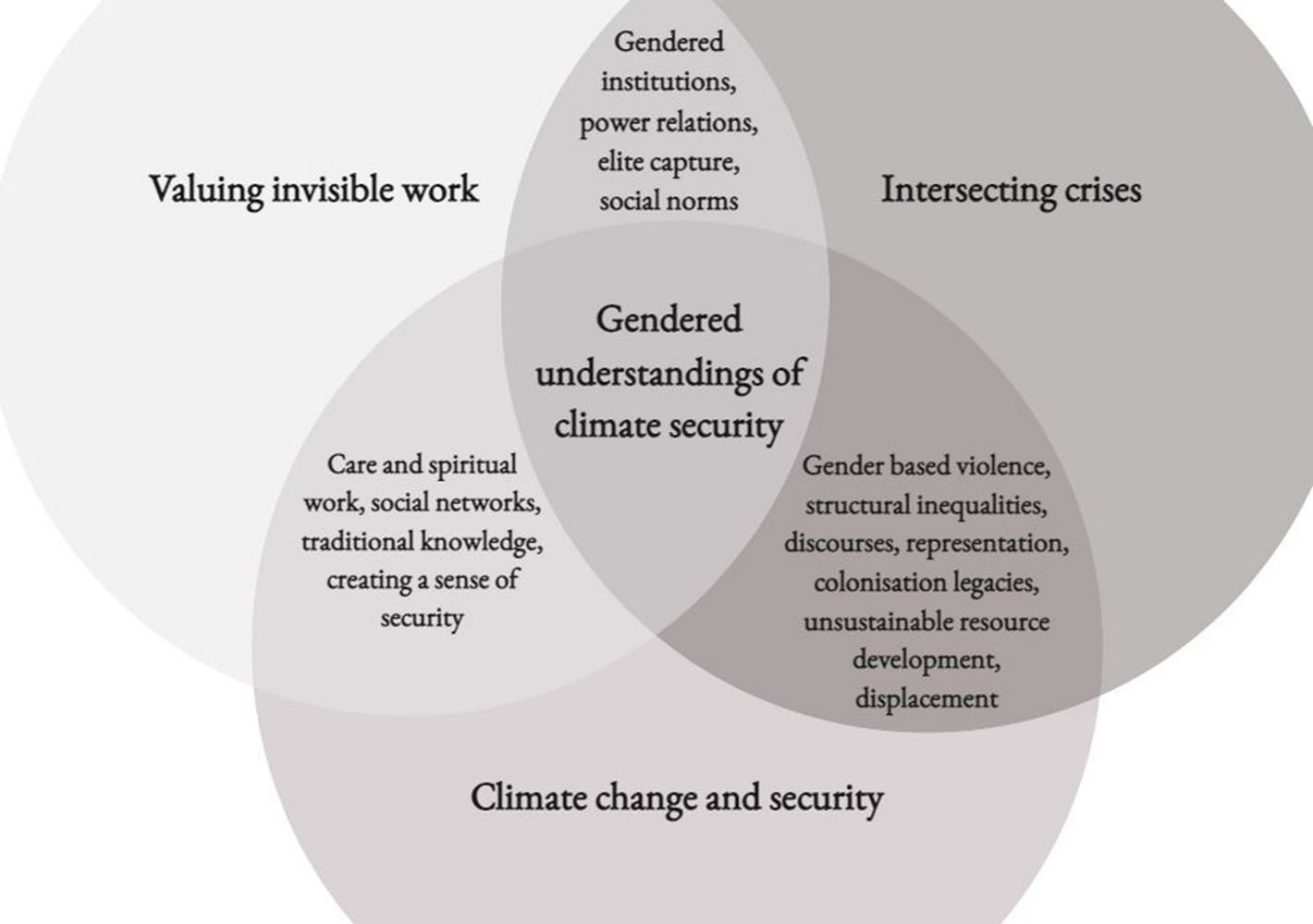
Existing empirical research that substantively analyses gender and links this with climate change and security has overlapping approaches and themes

The review aimed to categorise literatures according the spectrum in Fig. 1, however, in practice there is a significant overlap across the categories. This is likely because the gendered nature of institutions has led to a devaluing of women’s work, while efforts to consider the multiple impacts of intersecting crises are generally critiquing the masculine bias in decision making at local, national and regional levels. Therefore, ways in which gender, climate change and security were linked in existing scholarship are better represented as a Venn diagram to illustrate points of intersection, as represented in Fig. 2.

While there was significant overlap, literatures that did link gender, climate change and security (18) were categorised according to two broad aims. Firstly, a body of literature aimed to illustrate the consequences when gender inequalities in policy, resource management or decision making at local, national and regional levels interact with climate change and other crises (George 2014, 2016; Bhagwan Rolls and Rolls 2019; Roberts 2019; Davila et al. 2021). Secondly, a body of work is emerging that aims to document women’s contributions to security, particularly food and nutritional or livelihood security. These literatures are responding to a lack of visibility or recognition of the contributions that women’s ‘domestic’ labour makes to livelihoods, well-being and security (Grant 2019; Rabbit et al. 2019; Clissold et al. 2020; Sahai et al. 2021; Singh et al. 2022). The remaining articles were classified as having a predominant focus on the gendered nature of institutions, with consideration to intersecting crises (Lipset 2011; Mcleod 2018) or power relations (Buggy and McNamara 2016; Granderson et al. 2017; Ensor et al. 2018; Webb 2020; Mangubhai et al. 2021; Nakamura and Kanemasu 2022).

The first category of literature emphasises structural inequalities as a key source of insecurity, including unequal representation in politics or governance at national and community levels, poor spending levels on reproductive health, women’s over-representation in insecure work, engagement in sex work as a means of securing income or food, gendered access to resources and elevated levels of violence against women (George 2014, 2016; Mcleod et al. 2018; Bhagwan Rolls and Rolls 2019). Gendered land rights due to customary laws and practices were highlighted as a particular means of compromising security, particularly livelihood and food security (Mcleod et al. 2018). Women’s lack of involvement in decision-making over land use is apparent, including in matrilineal areas, the consequences of which become more pronounced during severe events such as prolonged drought (Roberts 2019).

Literatures also highlighted the double exposure of environmental degradation from resource extraction and climate change (Roberts 2019, p. 77) that cyclones can occur at the same time as a prolonged drought (Clissold et al. 2020), or the twin crises of COVID-19 with devastating cyclones (Davila et al. 2021; Mangubhai et al. 2021). Roberts’s (2019) ethnographic study illustrates the interaction of structural inequalities with poor regulation of resource extraction and climate change in depth. Climate change and environmental degradation pressures led to significant water, food and nutritional security issues colliding with changes in power differentials within community and households due to unequal benefit distribution from resource development. The twin pressures disadvantaged both men and women who were not the beneficiaries of resource extraction, and created significant workload burdens for women due to men’s absence for externally based employment at the same time that drought created increased difficulties in household work such as food preparation.

Inequalities in representation in national and regional forums were highlighted across some literatures. This scholarship responds to the politics of women’s exclusion from decision-making, or the performative rather than substantial commitment to gender equality declarations or the Women Peace and Security Agenda. Scholars advocated for the Women, Peace and Security agenda, which is typically a post-conflict tool, expand to include the impacts of climate change (George 2014, 2016; Bhagwan Rolls and Rolls 2019). Yet, this agenda has been sidelined rather than integrated and implemented within mainstream security forums. This scholarship also highlighted the impacts of militarisation for not only gender-based violence and sex work, but also the environmental degradation caused by military bases and weapons testing and that is exacerbated by climate change (George 2014, 2016; Bhagwan Rolls and Rolls 2019).

Literatures also used women’s experiences to illustrate that inclusion in unequal structures leads to a form of constrained agency. For example, women use their stereotypes as nurturers and carers, peacebuilders or environmental protectors to gain access to, legitimacy and voice in male-dominated forums where security is discussed and defined. Yet, this can come at a cost if these forums only recognise women’s agency where it relates to their essentialised roles, while dismissing or ignoring others. The effect contains women’s agency within unspoken boundaries (George 2016; Bhagwan Rolls and Rolls 2019). Constrained agency was evidenced across a number of articles, such as when women’s advocacy in market networks still had to be channelled through a male representative (Clissold et al. 2020); when sexual and reproductive health workers had to ‘make extra efforts to be brought in’ and advocate for their inclusion in health responses in post-disaster periods (Beek et al. 2021, p. 8); and when ‘speech norms’ (George 2014, p. 325) rewarded women who abstain from discussing the root causes of insecurities such as imperialism and militarisation when they engage with outside agencies such as UN or regional forums.

The second category of literatures responds to the lack of visibility and recognition of women’s work as a key contributor to public life. Women’s collective action and networks that bridge across communities at a national and regional level were highlighted as critical avenues for creating security in terms of advocacy and inclusion as well as in terms of food and livelihoods during times of climate change (Bhagwan Rolls and Rolls 2019; Clissold et al. 2020). Regional level networks such as femLINKpacific, the Fiji Women’s Crisis Centre, the Fiji Women’s Rights Movement, the Pacific arm of the Global Partnership for the Prevention of Armed Conflict (GPPAC Pacific), the Pacific Conference of Churches, the Pacific Centre for Peacebuilding and Transcend Oceania were highlighted as important forums for advocacy and representation for diverse voices that are not represented in parliaments (Bhagwan Rolls and Rolls 2019).

At a more local level, women’s networks through savings clubs for school fees, marketing collectives for agricultural produce, and handicrafts or church groups were important sources of food, economic and livelihood security, particularly in post-disaster periods. These networks had multiple material and well-being benefits such as enabling women’s innovation and entrepreneurship to develop diversified income sources; financial risk sharing or ‘grassroots insurance’; food sharing and information exchange; enabling collective action and advocacy; improving self-confidence and self-efficacy; facilitating assistance from non-affected to affected areas in post-disaster periods; and creating safe spaces and employing religion as a protective mechanism to ease distress during disaster (Gray et al. 2014; Clissold et al. 2020; Sahai 2021; Singh et al. 2022). At micro levels, family networks, support, sharing information and trust were major factors affecting disaster recovery (Singh et al. 2022). However, social capital or ‘risk sharing networks’ are less embedded in migrant, displaced, non-indigenous, squatter households or urban settlement communities who can be excluded from disaster responses due to racism or other forms of discrimination (Chandra and Gaganis 2016; Nakamura and Kanemasu 2022).

A small body of literature responds to women’s exclusion from data on fisheries catch or agriculture and aims to improve the recognition and valuing women’s contributions to food security in the context of climate change. Women’s traditional knowledges (Mcleod et al. 2018) and roles in gardening, agriculture, fisheries and markets (Eriksson et al. 2017; Roberts 2019; Rabbit et al. 2019; Clissold et al. 2020; Davila et al. 2021) were seen as key enablers for ongoing livelihoods, particularly during disaster recovery (Clissold et al. 2021; Singh et al. 2022). Authors also highlighted that women’s food security roles also provide other benefits for social stability. For example, women are the providers of nutrition to avoid stunted growth (Roberts 2019) or the key contributor to school fees, all roles that contribute in the long term to provide better prospects for youth employment and social stability (Davila et al. 2021).

This literature highlighted how social norms can place significant constraints on women’s agency. For example, studies highlighted that women’s diversified income sources are important to in post-disaster periods. Yet, women’s activity remained confined within or close by their village and conditional on fulfilling their expected roles such as caregiving or household work (Rabbit et al. 2019; Singh et al. 2022). Social norms constrain women’s mobility and prevent workload burden sharing between men and women, a situation exacerbated when men gain employment outside of communities (Roberts 2019). This workload is not limited to physical work, but also the psychological toll of care work, particularly in post-disaster periods, due to expectations that women will provide emotional support, care for family and household food supply. The psychological toll highlights the range of hidden costs that women experience when they shoulder the burdens of multiple roles (Clissold et al. 2020).

Lipset (2011) provided the only article within this sample to explicitly consider masculinities in the context of climate change. In an anthropological study of the Murik people who were affected by sea level rise and lacked power in negotiations with government for relocation, Lipset (2011) posits climate change, both as a speech act and material event, is a direct challenge to traditional forms of masculinity. Given literature in other contexts have highlighted ‘troubled masculinities’ as a potential driver of violence against women (Zimmer-Tamakoshi 2012), and there evidence of increased violence against women in post-disaster periods (Mcleod et al. 2018), the link between climate change and masculinity seems an important avenue of research.

Very few studies within the sample substantially considered how gender shapes experiences of migration, displacement or relocation. The exception to this is Wewerinke-Singh and Van Geelen (2018) who highlighted women’s choices were limited during evacuations due to gendered notions of protection. Evacuation led to long-term disadvantages in delayed re-establishment of gardens and prevention of forest overgrowth, and in disruptions to education for children. In other contexts, gendered spaces affected evacuation experiences, but lacked explicit treatment as a concept. For example, a lack of space and facilities in evacuation facilities created privacy concerns for women managing traditional taboos during menstruation (Chandra and Gaganis 2016) and elevated risks of sexual assault within these facilities (Wewerinke-Singh and Van Geelen 2018). In Webb’s (2020) study, women were initially denied access to a safe house to take shelter from an oncoming cyclone as they were menstruating, while it seems notions of masculinity prevented men from evacuating due to traditional beliefs that their house would fall if they were not present. This indicates that further work to explore the gendered dimensions of space, masculinity, femininity, evacuation and relocation are warranted.

## Conclusion

This review sought to understand how concepts of gender and security are given meaning and linked to climate change in empirical scholarship in the Pacific Islands Region. The review found that literatures are still dominated by simplified gender analysis, and that broad approaches to defining security are required to enable more critical engagement with the types of social issues and structural inequalities that influence experiences of security.

Despite the recognition that the impacts of climate change are gendered, in a region that is one of the most vulnerable to climate change across the globe, empirical scholarship that links climate change and security that considers gender is still limited to a small sample. Where literatures did mention gender, more than half was classified as an ‘adding women’ approach, most likely informed more by gender analysis toolkits than feminist analysis. Studies that consider gender as an intersectional form of marginalisation are important, particularly as urban settlement populations and displaced communities' experiences may lack the social capital and traditional knowledges that indigenous communities use to moderate vulnerabilities, and there is evidence of elite capture of climate adaptation or disaster recovery efforts. Few studies engaged critically with historical and contemporary influences on social hierarchies and gender relations such as colonisation, access to economic opportunities, the influence of a diaspora and internationally educated elite or urban rural divides. There is limited information on how climate change may also affect masculine forms of status and the consequences of this for gender relations or gender-based violence. While overall, women are overrepresented in terms of health, safety, social and economic disadvantage, this will be dependent on a range of factors that determine status. Greater engagement with the mechanisms that create marginalisation across gender, ethnic and socio-economic boundaries is needed.

Literatures that did link gender, climate change and security illustrate that simply including women or reporting on gender difference will not go far enough to address gendered experiences of climate change and security. Studies mostly focus on women’s experiences as a means of illuminating masculine bias in decision-making forums, policy or data collection. These studies highlight that greater attention needs to be paid to the interaction between climate change and gender-based violence, inequality, underrepresentation of women in decision making, poor governance of resource extraction, elite capture of climate change responses and gendered experiences of displacement. In addition, a critical lens is important to consider how dominant perspectives on risk and security may privilege particular values or worldviews over others due to taken-for-granted norms and power hierarchies in decision-making bodies. Women may be included in these forums, but experience significant constraints on their agency. While an important body of scholarship illustrates that responses to climate change have the potential to reinforce gendered hierarchies, most of this focus has been at community levels, with limited attention to the gendered nature of the broad-scale organisations with responsibilities for climate change and security responses. Continued questioning is needed to consider who constitutes the ‘human’ in understandings of security as this will have implications for perceptions of risk, priority issues and the shape of responses.

Approaches that engage less with gender as a category and more with shedding light on the power hierarchies in responses to climate change and security can highlight the devaluing of women’s roles and gendered hierarchies that influence who is perceived to have legitimacy in decision making. The emerging body of scholarship that aims to both broaden understandings of security and to bring greater visibility to women’s contributions to security has been important work to address the imbalance in these hierarchies. As these literatures point out, women’s roles in household food and water provision and care provide the foundations of peaceful and stable families and communities, yet these roles continue to be undervalued. At the same time, women’s agency is constrained by several factors: mobility, social norms, gendered violence, health, education, livelihood inequalities and gendered spaces, organisations and forums. Research that interrogates responses that may appear to be gender neutral to consider the assumptions, bias or what might be missing or overlooked is important to address these power hierarchies.

This review has several limitations due to its focus on academic scholarship and empirical studies, while a vast amount of knowledge and expertise is held outside of academic journals. However, it does indicate that greater links could be drawn between the critical understandings of gender and security to a changing biophysical environment. Greater research is needed that investigates the social–ecological–political economy nexus to draw attention to how climate change impacts, gender relations, spaces and institutions interact to construct experiences of security in the Pacific Islands Region.

## References

[CR1] Adger WN (2006). Vulnerability. Global Environmental Change.

[CR2] Adger WN (2011). Climate change, human well-being and insecurity. New Political Economy.

[CR3] Adger WN, Pulhin JM, Barnett J, Dabelko GD, Hovelsrud GK, Levy M, Oswald Spring Ú, Vogel CH, Field CB, Barros VR, Dokken DJ, Mach KJ, Mastrandrea MD, Bilir TE, Chatterjee M, Ebi KL (2014). Human security. Climate change 2014: impacts, adaptation, and vulnerability. Part A: global and sectoral aspects. Contribution of Working Group II to the Fifth Assessment Report of the Intergovernmental Panel on Climate Change.

[CR4] Ahmed S (2017). Living a feminist life.

[CR5] Aipira C, Kidd A, Morioka K, Filho WL (2017). Climate change adaptation in Pacific countries: Fostering resilience through gender equality. Climate change adaptation in Pacific countries: Fostering resilience and improving the quality of life.

[CR6] Arora-Jonsson S (2011). Virtue and vulnerability: Discourses on women, gender and climate change. Global Environmental Change.

[CR7] Asugeni R, Redman-MacLaren M, Asugeni J, Esau T, Timothy F, Massey P, MacLaren D (2019). A community builds a “bridge”: An example of community-led adaptation to sea-level rise in East Kwaio, Solomon Islands. Climate and Development.

[CR8] Baker K (2019). Pacific women in politics: Gender quota campaigns in the Pacific Islands.

[CR9] Balaei B, Wilkinson S, Potangaroa R (2019). Social capacities in fostering water supply resilience in Vanuatu. Disaster Prevention and Management.

[CR10] Barnett J, Boston J, Nel P, Righarts M (2009). Climate change and human security in the Pacific Islands: The potential for and limits to adaptation. Climate change and security planning for the future.

[CR11] Barnett J, O’Neill S (2010). Maladaptation. Global Environmental Change.

[CR12] Beek K, Drysdale R, Kusen M, Dawson A (2021). Preparing for and responding to sexual and reproductive health in disaster settings: Evidence from Fiji and Tonga. Reproductive Health.

[CR13] Bhagwan Rolls S, Rolls S, Davies SE, True J (2019). WPS and the Pacific Islands forum. The Oxford handbook of women, peace and security.

[CR14] Buggy L, McNamara KE (2016). The need to reinterpret “community” for climate change adaptation: A case study of Pele Island Vanuatu. Climate and Development.

[CR15] Carter G, Howard E (2020). Pacific women in climate change negotiations. Small States and Territories.

[CR16] Cauchi JP, Bambrick H, Moncada S, Correa-Velez I (2021). Nutritional diversity and community perceptions of health and importance of foods in Kiribati: A case study. Food Security.

[CR17] Chambers LE, Plotz R, Lui S, Aiono F, Tile T, Hiriasia D, Lloyd T, Ofa F (2021). Seasonal calendars enhance climate communication in the Pacific. Weather Society and Climate.

[CR18] Chand A, Taupo T, Amin SN, Watson D, Girard C (2020). Impact of natural disasters and climate change on national security in the Pacific. Case studies of Kiribati and Tuvalu. Mapping security in the Pacific: A focus on context, gender and organisational culture.

[CR19] Chandra A, Gaganis P (2016). Deconstructing vulnerability and adaptation in a coastal river basin ecosystem: A participatory analysis of flood risk in Nadi, Fiji Islands. Climate and Development.

[CR20] Cleasby N, Schwarz A, Phillips M, Paul C, Pant J, Oeta J, Pickering T, Meloty A (2014). The socio-economic context for improving food security through land based aquaculture in Solomon Islands: A peri-urban case study. Marine Policy.

[CR21] Clissold R, Westoby R, McNamara K (2020). Women as recovery enablers in the face of disasters in Vanuatu. Geoforum.

[CR22] Cornwall A, Harrison E, Whitehead A, Cornwall A, Harrison E, Whitehead A (2007). Introduction: Feminisms in development: Contradictions, contestations and challenges. Feminisms in development: Contradictions, contestations and challenges.

[CR23] Crichton RN, Esteban M, Onuki M (2020). Understanding the preferences of rural communities for adaptation to 21st-century sea-level rise: A case study from the Samoan islands. Climate Risk Management.

[CR24] Davila F, Bourke RM, McWilliam A, Crimp S, Robins L, van Wensveen M, Alders R, Robyn G (2021). COVID-19 and food systems in Pacific Island Countries, Papua New Guinea, and Timor-Leste: Opportunities for actions towards the sustainable development goals. Agricultural Systems.

[CR25] Day J, Wewerinke-Singh M, Price S (2021). Eviction is not a disaster. Development Policy Review.

[CR26] Dellmuth L, Gustafsson MT, Bremberg N, Mobjork M (2018). Intergovernmental organizations and climate security: Advancing the research agenda. Wires Climate Change.

[CR27] de Jong Oudraat, C. and M. E. Brown. 2022. Gender, climate change and security: making the connections. https://www.wilsoncenter.org/article/gender-climate-change-and-security-making-connections Accessed 6 Feb 2022.

[CR28] Detraz N (2015). Environmental security and gender.

[CR29] Detraz N, MacGregor S (2017). Gender and environmental (in)security. From climate conflict to ecosystem instability. Routledge handbook of gender and environment.

[CR30] Djoudi H, Locatelli B, Vaast C, Asher K, Brockhaus M, Sijapati BB (2016). Beyond dichotomies: Gender and intersecting inequalities in climate change studies. Ambio.

[CR31] Elmhirst R, Perreault T, Bridge G, McCarthy J (2015). Feminist political ecology. The Routledge handbook for political ecology.

[CR32] Enloe C (2014). Banana, beaches and bases: Making feminist sense of international politics.

[CR33] Ensor JE, Abernethy KE, Hoddy ET, Aswani S, Albert S, Vaccaro I, Benedict JJ, Beare DJ (2018). Variation in perception of environmental change in nine Solomon Islands communities: Implications for securing fairness in community-based adaptation. Regional Environmental Change.

[CR34] Eriksen SH, Nightingale AJ, Eakin H (2015). Reframing adaptation: The political nature of climate change adaptation. Global Environmental Change.

[CR35] Eriksen SH, Schipper ELF, Scoville-Simonds M, Vincent K, Adam HN, Brooks N, Harding B, Khatri D (2021). Adaptation interventions and their effect on vulnerability in developing countries: Help, hindrance or irrelevance?. World Development.

[CR36] Eriksson H, Albert J, Albert S, Warren R, Pakoa K, Andrew N (2017). The role of fish and fisheries in recovering from natural hazards: Lessons learned from Vanuatu. Environmental Science and Policy.

[CR37] Farbotko C, Klöck C, Fink M (2019). Climate change displacement: Towards ontological security. Dealing with climate change on small islands: Towards effective and sustainable adaptation?.

[CR38] Gemenne F, Barnett J, Adger WN, Dabelko G (2014). Climate and security: Evidence, emerging risks, and a new agenda. Climatic Change.

[CR39] George N (2014). Promoting women, peace and security in the Pacific Islands: Hot conflict/slow violence. Australian Journal of International Affairs.

[CR40] George N (2016). Institutionalising Women, Peace and Security in the Pacific Islands: Gendering the 'architecture of entitlements'?. International Political Science Review.

[CR41] Granderson AA (2017). The role of traditional knowledge in building adaptive capacity for climate change: Perspectives from Vanuatu. Weather, Climate, and Society.

[CR42] Grant C (2019). Climate justice and cultural sustainability: The case of etëtung (Vanuatu women’s water music). The Asia Pacific Journal of Anthropology.

[CR43] Gray BJ, Duncanb S, Kirkwood J, Walton S (2014). Encouraging sustainable entrepreneurship in climate-threatened communities: A Samoan case study. Entrepreneurship and Regional Development.

[CR44] Hansen L, Shepherd L (2014). Ontologies, epistemologies, methodologies. Gender matters in global politics: A feminist introduction to international relations.

[CR45] Hoogensen G, Stuvøy K (2006). Gender, resistance and human security. Security Dialogue.

[CR46] Pörtner H-O, Roberts DC, Poloczanska ES, Mintenbeck K, Tignor M, Alegría A, Craig M, Langsdorf S, IPCC (2022). Summary for policymakers. Climate change 2022: impacts, adaptation and vulnerability. Contribution of Working Group II to the Sixth Assessment Report of the Intergovernmental Panel on Climate Change.

[CR47] Jolly M, Sawer M, Jenkins F, Downing K (2020). Moving beyond disciplinary boundaries to respond to climate change. How gender can transform the social sciences. Innovation and impact.

[CR48] Kaijser A, Kronsell A (2014). Climate change through the lens of intersectionality. Environmental Politics.

[CR49] Klöck C, Nunn PD (2019). Adaptation to climate change in small island developing states: A systematic literature review of academic research. Journal of Environment and Development.

[CR50] Kronsell A, Ackerly B, Stern M, True J (2010). Methods for studying silences: Gender analysis in institutions of hegemonic masculinity. Feminist methodologies for international relations.

[CR51] Kronsell A, Davies S, True J (2019). WPS and climate change. The Oxford handbook of women peace and security.

[CR52] Lawless S, Cohen PJ, Mangubhai S, Kleiber D, Morrison TH (2021). Gender equality is diluted in commitments made to small-scale fisheries. World Development.

[CR53] Lawless S, Cohen PJ, McDougall C, Mangubhai S, Song AM, Morrison TH (2022). Tinker, tailor or transform: Gender equality amidst social-ecological change. Global Environmental Change.

[CR54] Lipset D (2011). The tides: Masculinity and climate change in coastal Papua New Guinea. Journal of the Royal Anthropological Institute.

[CR55] Malherbe W, Sauer W, Aswani S (2020). Social capital reduces vulnerability in rural coastal communities of Solomon Islands. Ocean & Coastal Management.

[CR56] Mangubhai S, Nand Y, Reddy C, Jagadish A (2021). Politics of vulnerability: Impacts of COVID-19 and Cyclone Harold on Indo-Fijians engaged in small-scale fisheries. Environmental Science & Policy..

[CR57] Mangubhai S, Lawless S, Cowley A, Mangubhai JP, Williams M (2022). Progressing gender equality in fisheries by building strategic partnerships with development organisations. World Development.

[CR58] McDonald M (2021). Ecological security: Climate change and the construction of security.

[CR59] McLaughlin P, Dietz T (2008). Structure, agency and environment: Toward an integrated perspective on vulnerability. Global Environmental Change.

[CR60] Mcleod E, Arora-Jonsson S, Masuda YJ, Bruton-Adams M, Emaurois CO, Gorong B, Hudlow CJ, James R (2018). Raising the voices of Pacific Island women to inform climate adaptation policies. Marine Policy.

[CR61] McNamara KE, Buggy L (2017). Community-based climate change adaptation: A review of academic literature. Local Environment.

[CR62] Mohanty CT (2003). Feminism without borders: Decolonizing theory, practicing solidarity.

[CR63] Mycoo M, Wairiu M, Campbell D, Duvat V, Golbuu Y, Maharaj S, Nalau J, Nunn P, Pörtner HO, Roberts DC, Tignor M, Poloczanska ES, Mintenbeck K, Alegría A, Craig M, Langsdorf S (2022). Small Islands. Climate change 2022: Impacts, adaptation and vulnerability. Contribution of Working Group II to the Sixth Assessment Report of the Intergovernmental Panel on Climate Change.

[CR64] Nagel J (2012). Intersecting identities and global climate change. Identities.

[CR65] Nakamura N, Kanemasu Y (2022). A minority group's response to a severe climatic event: A case study of rural Indo-Fijians after Tropical Cyclone Winston in 2016. Disasters.

[CR66] Nef DP, Neneth D, Dini P, Carmenzo R, Kruetli P (2021). How local communities attribute livelihood vulnerabilities to climate change and other causes: A case study in North Vanuatu. Climatic Change.

[CR67] Nelson S, Abimbola S, Mangubhai S, Jenkins A, Jupiter S, Naivalu K, Naivalulevu V, Negin J (2021). Understanding the decision-making structures, roles and actions of village-level water committees in Fiji. International Journal of Water Resources Development.

[CR68] Oswald-Spring U, Brauch HG, Oswald Spring U, Grin J, Mesjasz C, Kameri-Mbote P, Behara NC, Chourou B, Krummenacher H (2009). A HUGE gender security approach: Towards human, gender and environmental security. Facing global environmental change environmental, human, energy, food, health and water security concepts.

[CR69] Pacific Islands Forum Secretariat. 2018. Boe Declaration on Regional Security, Retrieved 10 August, 2022, from https://www.forumsec.org/2018/09/05/boe-declaration-on-regional-security/

[CR70] Pacific Islands Forum Secretariat. 2022. Communique of the 51^st^ Pacific Islands Forum Leaders Meeting. Retrieved 10 August, 2022, from https://www.forumsec.org/2022/07/17/report-communique-of-the-51st-pacific-islands-forum-leaders-meeting/

[CR71] Pacific Women. 2020. Ending violence against women. Retrieved 11 August, 2022, from https://pacificwomen.org/our-work/focus-areas/ending-violence-against-women/

[CR72] Pascoe S, Dressler W, Minnegal M (2021). Storytelling climate change. Causality and temporality in the REDD plus regime in Papua New Guinea. Geoforum.

[CR73] Peterson VS (2005). How the meaning of gender matters in political economy. New Political Economy.

[CR74] Pickering C, Byrne J (2014). The benefits of publishing systematic quantitative literature reviews for PhD candidates and other early-career researchers. Higher Education Research & Development.

[CR75] PRISMA. 2020. PRISMA Checklist. Retrieved 4 January 2022, from http://prisma-statement.org/PRISMAStatement/Checklist

[CR76] Rabbitt S, Lilley I, Albert S, Tibbetts IR (2019). What's the catch in who fishes? Fisherwomen's contributions to fisheries and food security in Marovo Lagoon Solomon Islands. Marine Policy.

[CR77] Rasheed AA (2022). Small Island Developing States and climate securitisation in international politics: Towards a comprehensive conception. Island Studies Journal.

[CR78] Ratuva S (2019). Contested terrain, reconceptualising security in the Pacific.

[CR79] Rey T, Le Loic D, Leone F, Gilbert D (2017). An integrative approach to understand vulnerability and resilience post-disaster. Disaster Prevention and Management.

[CR80] Robbins P (2012). Political ecology: A critical introduction.

[CR81] Roberts J (2019). 'We live like this': Local inequalities and disproportionate risk in the context of extractive development and climate change on New Hanover Island, Papua New Guinea. Oceania.

[CR82] Robinson F (2011). The ethics of care: A feminist approach to human security.

[CR83] Rocheleau D, Thomas-Slayter B, Wangari E, Rocheleau D, Thomas-Slayter B, Wangari E (1996). Gender and environment. A feminist political ecology perspective. Feminist political ecology, global issues and local experiences.

[CR84] Runyan AS, Peterson VS (2015). Global gender issues in the new millennium.

[CR85] Rydstrom H, Kinvall C, Kinvall C, Rydstrom H (2019). Introduction. Climate hazards, disasters and gender ramifications.

[CR86] Sahai S, Tabe T, Ryle J, Luetz J, Nunn P (2021). Cyclone Winston: Catholic women’s faith and agency in a coastal village in Fiji. Beyond belief. Opportunities for faith-engaged approaches to climate-change adaptation in the Pacific Islands.

[CR87] Savage A, Schubert L, Huber C, Bambrick H, Hall N, Bellotti B (2020). Adaptation to the climate crisis: Opportunities for food and nutrition security and health in a Pacific small island state. Weather, Climate, and Society.

[CR88] Senimoli AN, Tabe T, Jacot Des Combes H (2020). Influence of socio-cultural factors on community disaster response during TC Winston: A case study of Burenitu Village, Fiji. International Journal of Safety and Security Engineering.

[CR89] Shani G (2017). Human security as ontological security: A post-colonial approach. Postcolonial Studies.

[CR90] Singh P, Tabe T, Martin T (2022). The role of women in community resilience to climate change: A case study of an Indigenous Fijian community. Women's Studies International Forum.

[CR91] Sovacool BK, Valentine SB, Bambawale MJ, Brown MA, Cardoso T, Nurbek S, Suleimenova G, Li J (2012). Exploring propositions about perceptions of energy security: An international survey. Environmental Science and Policy.

[CR92] Sundberg J, Richardson D, Castree N, Goodchild MF, Kobayashi A, Liu W, Marston RA (2017). Feminist political ecology. International Encyclopedia of Geography: People, the Earth, environment and technology.

[CR93] Tangney P, Nettle C, Clarke B, Newman J, Star C (2021). Climate security in the Indo-Pacific: A systematic review of governance challenges for enhancing regional climate resilience. Climatic Change.

[CR94] Tanyag M (2018). Depleting fragile bodies: The political economy of sexual and reproductive health in crisis situations. Review of International Studies.

[CR95] Tanyag, M. 2020. From alarm bells to background noise? The role of gender in risk mapping, analysis and response in the Asia Pacific Region. In *Gender, climate and security. Sustaining inclusive peace on the frontlines of climate change*, eds. UNEP, UN Women, DPPP and UNDP, 34–35.

[CR96] Tanyag M, True J, Kinvall C, Rydstrom H (2019). Gender-responsive alternatives on climate change from a feminist standpoint. Climate hazards, disasters and gender ramifications.

[CR97] Teaiwa TK (2021). Sweat and salt water. Selected works.

[CR98] Teariki MA, Tiatia R, O'Sullivan K, Puloka V, Signal L, Shearer I, Howden-Chapman P (2020). Beyond home: Exploring energy poverty among youth in four diverse Pacific Island states. Energy Research and Social Science.

[CR99] Tickner JA, Ackerly BA, Stern M, True J (2006). Feminism meets international relations: Some methodological issues. Feminist methodologies for international relations.

[CR100] True J (2012). The political economy of violence against women.

[CR101] Underhill-Sem Y, Chan Tung A, Marsters E, Eftonga Pene S (2016). Gender research in the Pacific 1994–2014: Beginnings.

[CR102] UNDP 2020. United Nations launches pioneering Climate Security Project in the Pacific supported by UN SG’s Peacebuilding Fund. Accessed 7 July, 2021, from https://www.undp.org/pacific/press-releases/united-nations-launches-pioneering-climate-security-project-pacific-supported-un-sgs-peacebuilding-fund

[CR103] UNEP, UN women, DPPA and UNDP. 2020. Gender, climate and security. Sustaining inclusive peace on the frontlines of climate change. Retrieved 2 August 2022 from https://wedocs.unep.org/bitstream/handle/20.500.11822/32638/GCS.pdf?sequence=1&isAllowed=y

[CR104] van der Ploeg J, Meshach S, Hugh G, Tessa M, Hampus E (2020). Sinking islands, drowned logic; Climate change and community-based adaptation discourses in Solomon Islands. Sustainability.

[CR105] Webb J (2020). What difference does disaster risk reduction make? Insights from Vanuatu and tropical cyclone Pam. Regional Environmental Change.

[CR106] Weir T, Filho WL (2020). Adaptation in small islands Research themes and gaps. Managing climate change adaptation in the Pacific Region.

[CR107] Wentworth C (2020). Unhealthy aid: Food security programming and disaster responses to Cyclone Pam in Vanuatu. Anthropological Forum.

[CR108] Westoby R, McNamara KE, Kumar R, Nunn PD (2020). From community-based to locally led adaptation: Evidence from Vanuatu. Ambio.

[CR109] Wewerinke-Singh M, Van Geelen T (2018). Protection of climate displaced persons under international law: A case study from Mataso Island. Vanuatu. Melbourne Journal of International Law.

[CR110] Wonders N, Fitz-gibbon K, Walklate S, McCulloch J, Maher J (2018). Climate change, the production of gendered insecurity and intimate partner violence. Intimate partner violence, risk and security—securing women’s lives in a global world.

[CR111] Zimmer-Tamakoshi L, Jolly M, Stewart C (2012). Troubled masculinities and gender violence in Melanesia. Engendering violence in Papua New Guinea.

